# Mental health across two years of the COVID-19 pandemic: a 5-wave longitudinal study in Germany

**DOI:** 10.3389/fpsyt.2023.1229700

**Published:** 2023-08-08

**Authors:** Maxi Weber, Sebastian Burchert, Marit Sijbrandij, Martina Patanè, Irene Pinucci, Babette Renneberg, Christine Knaevelsrud, Sarah Schumacher

**Affiliations:** ^1^Department of Education and Psychology, Clinical Psychology and Psychotherapy, Freie Universität Berlin, Berlin, Germany; ^2^Department of Education and Psychology, Clinical Psychological Intervention, Freie Universität Berlin, Berlin, Germany; ^3^Department of Clinical, Neuro-, and Developmental Psychology, Amsterdam Public Health Institute and WHO Collaborating Center for Research and Dissemination of Psychological Interventions, Vrije Universiteit, Amsterdam, Netherlands; ^4^Department of Human Neurosciences, Sapienza University of Rome, Rome, Italy; ^5^Clinical Psychology and Psychotherapy, Institute for Mental Health and Behavioral Medicine, HMU Health and Medical University, Potsdam, Germany

**Keywords:** mental health, depression, anxiety, pandemic, longitudinal

## Abstract

The COVID-19 pandemic has been negatively associated with mental health. However, little is known about the temporal dynamics of mental health in the longer term of the pandemic. We aimed to investigate symptom levels and changes of depression, anxiety, posttraumatic stress, and loneliness spanning two years of the pandemic; and to examine associated risk factors. This five-wave, longitudinal online study from May 2020 to April 2022 included 636 adults (M*_age_* = 39.5 years, *SD* = 16.11; 84.1% female) from the German general population who completed the international COVID-19 Mental Health Survey. Symptoms of anxiety (Generalized Anxiety Disorder-7; GAD-7), depression (Patient Health Questionnaire-9; PHQ-9), posttraumatic stress (PTSD Checklist for DSM-5; PCL-5), and loneliness (“*Do you feel lonely?”*) were assessed using mixed-effects models. Associations with anxiety and depressive symptoms were examined with having children, student status, financial worries, contamination fear, and loneliness. PHQ-9, GAD-7, PCL-5, and loneliness scores overall decreased throughout the two-year period of the pandemic but exhibited an increase during two national lockdowns. Controlled for significant associations with female gender and younger age, increased PHQ-9 and GAD-7 scores were associated with contamination fear, financial worries, and loneliness. No associations were found with having children and student status. Symptoms of depression, anxiety, posttraumatic stress, and loneliness decreased over time but varied along with the dynamics of the pandemic. Longitudinal monitoring of mental health in vulnerable subgroups is required, especially those of younger age, females, and the financially insecure.

## 1. Introduction

The COVID-19 pandemic is characterized as a long-term, multifaced stressor for mental health (1). Mental health responses to the first months of the pandemic indicated high rates of anxiety, depression, and related symptoms in the general population across the globe, albeit largely based on cross-sectional studies [see for summaries (2–8)]. During the early pandemic phase, systematic evidence from longitudinal studies showed a slight to moderate increase in mental burden compared to pre-pandemic years (9–14), which decreased slightly with time and the easing of pandemic containment measures (10, 13). In a recent umbrella review of systematic reviews and meta-analyses (15), this increase during the pandemic was generally larger and longer lasting for symptoms of depression than for anxiety.

A few longitudinal studies investigating changes in mental health covered the first year of the pandemic with multiple infection waves and lockdown periods. Four studies using data from the international COVID-19 Mental Health Survey (COMET) – a prospective online study assessing the mental health of the general population in 14 countries – examined symptoms of anxiety, depression, posttraumatic stress, and loneliness up to April 2021 among the adult population (16–18), and subgroups with chronic medical conditions (19). Gémes et al. (16) investigated prevalence rates of depressive and anxiety symptoms, accounting for differences in existing mental disorders and migrations status across Australia and six European countries including Germany. Initial prevalence rates varied between countries and changes in prevalence rates over time were rather small. In most countries, no differences in time trends were observed with regard to migration status or an existing mental health condition. In Germany, individuals with a prior mental disorder showed a decreasing rate of anxiety over time; in Spain, this association held true for a decreased rate of depression (16). Two other COMET studies among a subset of French participants showed fluctuating mean scores of depression, anxiety, posttraumatic stress symptoms (17), and distinct trajectories of loneliness (18) during the pandemic.

Additional studies showed temporal associations between psychological distress and the later pandemic waves and lockdowns. Two studies reported increasing symptoms of depression, anxiety, and distress during the second and third pandemic wave compared to pre-pandemic and earlier pandemic phases among the UK (20) and Argentinian populations (21). In Germany, one study indicated increased symptoms of anxiety and depression during the second pandemic wave and lockdown compared to pre-pandemic measures, but decreasing symptoms compared to the first pandemic wave and lockdown (22). In another study among the German adult population, anxiety and depressive symptoms were overall declining with the duration of the pandemic (23). However, additional peaks were evident during the second lockdown period, as well as subsequent decreases in the easing phase.

Studies also revealed risk factors associated with increased symptoms of depression and anxiety during the pandemic, including female gender and younger age (13, 16–20). Increased symptomatology was more prevalent among student groups and individuals living with children compared to the general population (2, 13, 20). Similarly, economic factors such as loss of job, financial insecurity, or lower socioeconomic status were associated with increased mental burden during the pandemic (21, 24). Among psychological outcomes, loneliness and fear of contamination have been reported as risk factors for mental health (17–19). Yet, less is known about the associations with changes in mental health in the longer term of the pandemic.

**Figure 1:**
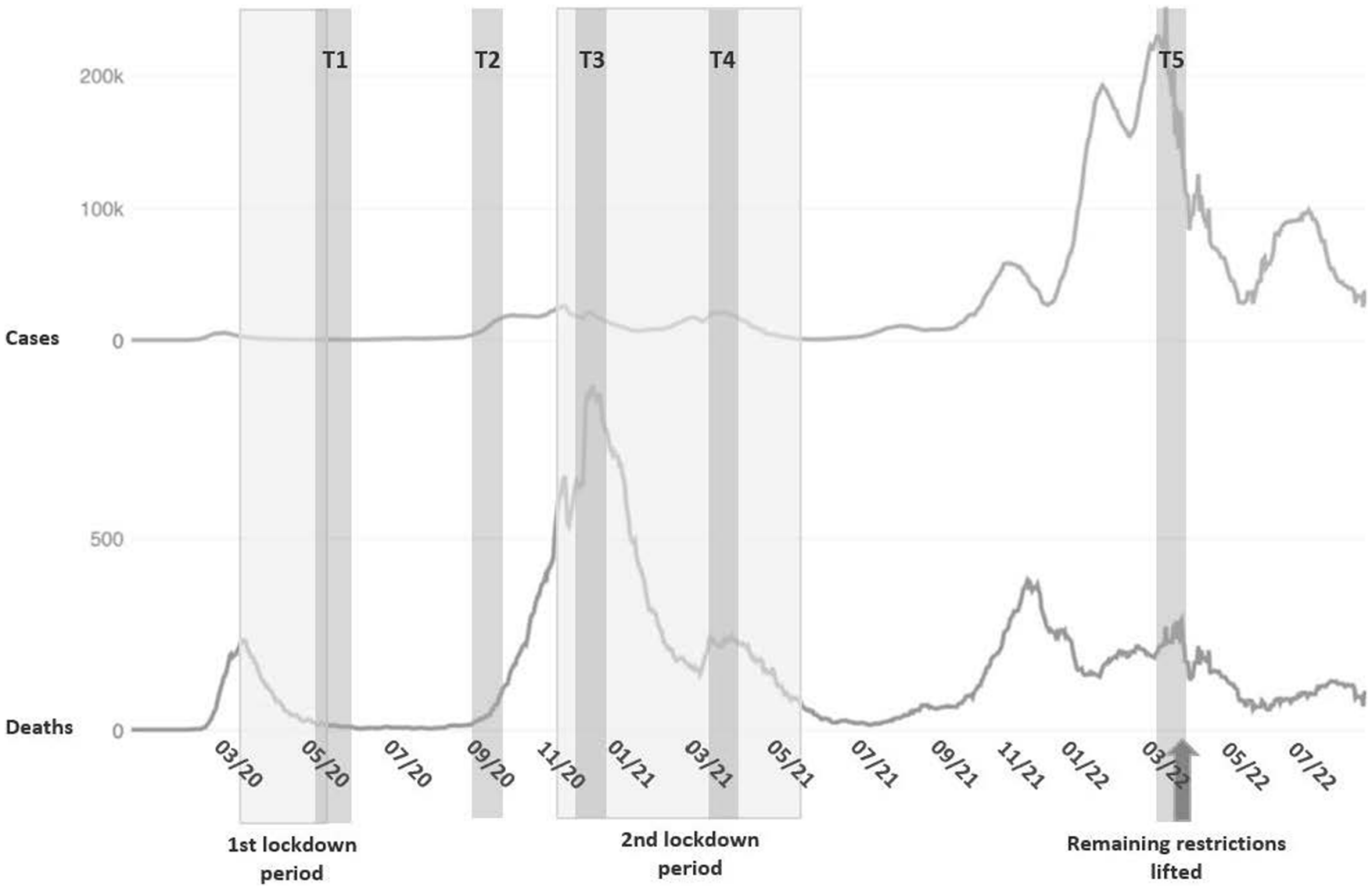
Data collection period, Covid-19 cases, and related death rates in Germany [COVID-19 Data Repository, Johns Hopkins University, 2022 (25)]. The German government initiated the first lockdown on March 23, 2020 (26), and stepwise relaxed preventive measures on May 5, 2022 onwards (27). The second lockdown period started on November 2, 2020 (28) with tightened restrictions on December 16, 2020 (29), which were gradually relaxed in mid-May 2022 onwards in the respective federal state. During the most restrictive lockdown phases, schools, universities, restaurants, and most shops were closed, and social distancing was mandatory in groups of maximum four people. Between March 19 and April 2, 2022 all the remaining restrictions in the federal states were lifted except for wearing masks in hospitals and public transport (30).

As most previous studies have focused on the first year of the pandemic revealing heterogeneous results, long-term monitoring of mental health seems crucial, including symptoms of anxiety, depression, loneliness, and posttraumatic stress. To date, studies are lacking that capture the longer-term dynamics of different mental health facets up to 2022 and contrast multiple lockdown and easing phases of the pandemic. This would allow for a more nuanced depiction of mental health trajectories during the two-year pandemic and its potential consequences. The present, 5-wave longitudinal study from May 2020 to April 2022 aims to add insight into the mental health changes during the COVID-19 pandemic in Germany. First, we aimed to investigate symptoms of depression, anxiety, posttraumatic stress, and loneliness in the German adult population as a function of time, while accounting for inter-individual differences. We expected a deterioration in mental health during the two long-lasting lockdown periods in Germany (Figure 1). Second, we aimed to identify risk factors for mental health in the longer term of the pandemic. Specifically, we examined whether anxiety and depressive symptoms and changes across time were negatively associated with the factors having children, being a student, as well as higher levels of financial worries, contamination fear, and loneliness, respectively.

## 2. Methods

We followed the Strengthening the Reporting of Observational Studies in Epidemiology (STROBE) checklist in reporting this observational study (Supplementary Table S1) (31). The study protocol is available at: https://osf.io/bgtsf/.

### 2.1. Study design and participants

The study data originate from the online longitudinal COMET study measuring the mental health of the general population in 13 countries during two years of the COVID-19 pandemic (16, 17). The COMET study was approved by the Scientific and Ethical Review Board of Vrije Universiteit Amsterdam (VCWE-2020-077) and by the local Ethical Review Board of the Department of Education and Psychology at Freie Universität Berlin, Germany (023/2020). As random sampling was not possible, the study population was recruited through mailing lists and social media platforms at the beginning of the pandemic. Eligible participants were at least 18 years of age, provided online informed consent, and showed sufficient knowledge of the language in each of the 13 participating countries, respectively. In this study we report on *n* = 636 individuals from the general population living in Germany, who were surveyed in May–June 2020 (T1) and in four subsequent waves up to March–April 2022 during the pandemic (T1: *n* = 636, T2: *n* = 462, T3: *n* = 449, T4: *n* = 426, and T5: *n* = 216). Figure 1 details all assessment time points combined with the pandemic situation in Germany during the survey period. Of the 636 participants, 105 (16.5%) took part in one, 76 (11.9%) in two, 72 (11.3%) in three, 206 (32.4%) in four, and 177 (27.8%) in all five assessments.

### 2.2. Measurements

The survey included baseline items on sociodemographic data such as age and gender, the economic and living situation, the presence of a current mental disorder (Table 1), and care-work related items such as the perceived impact of school or daycare closures. Covid-19 infection rates and the implemented lockdown measures were measured at each assessment. Mental health was surveyed repeatedly via depressive and anxiety symptoms, posttraumatic stress symptoms, and perceived loneliness.

**Table 1:** Characteristics of participants at baseline (T1, May–June 2020) and at the latest follow-up (T5, April–May 2022).

	T1 (*n* = 636) *n* (%)	T5 (*n* = 216) *n* (%)
Initial age		
Mean (SD)^a^	39.53 (16.11)	40.48 (17.03)
Median	37.0	41.0
Missing	1	1
Gender		
Female	535 (84.1%)	176 (81.5%)
Male	85 (13.4%)	31 (14.4%)
Diverse	15 (2.4%)	9 (4.2%)
Missing	1 (0.2%)	0 (0%)
Nationality		
German	544 (85.5%)	197 (91.2%)
Other	92 (8.8%)	7 (3.2%)
Missing	36 (5.7%)	12 (5.6%)
Relationship status		
Partnership/Married	376 (59.1%)	136 (63.0%)
Not in a relationship	257 (40.4%)	78 (36.1%)
Missing	3 (0.5%)	2 (0.9%)
Having children		
Yes	228 (35.8%)	85 (39.4%)
No	398 (62.6%)	131 (60.6%)
Missing	10 (1.6%)	0 (0%)
Living situation		
Alone	186 (29.2%)	69 (31.9%)
With others	445 (70.0%)	144 (66.7%)
Missing	5 (0.8%)	3 (1.4%)
Educational level		
Elementary school or no former education	64 (10.1%)	14 (6.5%)
Secondary school	278 (43.7%)	99 (45.8%)
Higher education	276 (43.4%)	98 (45.4%)
Missing	18 (2.8%)	5 (2.3%)
Covid-19 infection^b^		
Yes, confirmed by formal test	4 (0.6%)	68 (31.5%)
Most likely, but not confirmed	45 (7.1%)	18 (8.3%)
No	585 (92.0%)	128 (59.3%)
Missing	2 (0.3%)	2 (0.9%)
Prior diagnosed mental disorder		
Yes	231 (36.3%)	84 (38.9%)
No	405 (63.7%)	131 (60.6%)
Missing	0 (0%)	1 (0.5%)

a SD, standard deviation.

b Participants were screened for current Covid-19 infection at all five assessments.

Depressive and anxiety symptoms during the last two weeks were assessed with the *Patient Health Questionnaire–9* [PHQ-9, (32, 33)] *and the Generalized Anxiety Disorder Scale–7* [GAD-7, (34, 35)]. Each scale is rated on a 4-point Likert scale ranging from 0 “not at all” to 3 “nearly every day.” Sum scores range from 0 to 27 for the PHQ-9 and from 0 to 21 for the GAD-7, while scores of ≥10 indicate moderate-to-severe depressive or anxiety symptoms, respectively. In this study, Cronbach’s α ranged between 0.90 and 0.92 for the two scales, indicating good reliability (Supplementary Tables S4, S5).

Posttraumatic stress symptoms in the previous month were assessed with the validated 4-item version of the *PTSD Checklist for DSM-5* [PCL-5, (36, 37)]. Items are rated on a 5-point Likert scale ranging from 0 “not at all” to 4 “extremely.” Mean scores range between 0 and 20, with higher scores indicating a higher load of posttraumatic stress symptoms (Cronbach’s *α* 0.79–0.84, Supplementary Table S5). Current loneliness was rated with the single-item “*Do you feel lonely*?” on a 5-point Likert scale ranging from 1 “never” to 5 “frequently,” which proved to be a reliable and valid measure compared to multi-item loneliness scales (38). Higher scores relate to higher levels of perceived loneliness.

### 2.3. Statistical analysis

Descriptive analyses and mixed effects models using the MIXED procedures were calculated in IBM SPSS Statistics (Version 29) (39). Mixed effects modeling was applied to analyze the mental health symptoms and predictors of symptom load and change during the pandemic. In these models, multiple repeated measures are nested within individuals. In comparison to traditional methods (e.g., repeated measures analyses of variance), mixed models provide less biased variance components in incomplete and unbalanced data using all available data (40, 41), and do not require multiple imputation of missing values (42). We derived separate mixed effects models with random intercepts and slopes for each of the four outcome variables: depressive symptoms (PHQ-9), anxiety symptoms (GAD-7), posttraumatic stress symptoms (short-form PCL-5), and loneliness (single item: “*Do you feel lonely?*”). First, a null model was built to determine the intraclass correlation coefficient (ICC) independently for each outcome (Model 0), as in all following models. The ICCs for symptoms of depression, anxiety, posttraumatic stress, and loneliness suggested that 77.9%, 74.8%, 71.1%, and 67.4% of the total variation in symptoms was due to inter-individual differences, respectively. Second, an unconditional random-coefficients model was computed, in which *time* (T1-T5) was modeled as a time-structured predictor to account for irregularly spaced measurement occasions (41) (Model 1). The baseline (T1) value was set to zero and the consecutive months of follow-up divided by 12 were entered into the model. Third, this model was expanded by testing polynomial functions to adequately assess changes over time (Model 2) (43). Therefore, we gradually included linear, quadratic, and cubic time trends in the model with unstructured covariances differing at each time point. In the final unconditional model (Model 3), fixed effects were time, time-squared, and time-cubed curves, representing the mean change for the entire sample. The random effect was time, thereby accounting for the variation of individual changes around this group mean.

In addition to the unconditional model, we fitted a conditional model to study changes over time adjusted for the time-invariant covariates initial age (at T1) and gender (0 = male, 1 = female) for each outcome variable (Model 4). Both covariates were treated as continuous variables and centered by their means. For depressive symptoms and anxiety symptoms, we additionally tested whether pre-specified predictor variables adjusted for initial age and gender were associated with the initial symptom load and with changes in symptom load over time. Each mean-centered predictor was examined individually, that is, having children (0 = no, 1 = yes), student status (0 = no, 1 = yes), initial financial worries (T1; single item: “*During the past 4 weeks, have you worried about your financial situation?*”), initial contamination fear [T01; *Padua Inventory-Revised, Contamination obsessions and Washing compulsions subscale* (44, 45)], and loneliness as a time-varying variable (T1-T5; *“Do you feel lonely?”*; Models 5–10). All predictors and time*predictor interactions were entered as fixed effects, while loneliness was further examined as random effect.

To test the explained variance over added complexity we compared model fits using the Likelihood Ratio Test, and indices of the Bayesian Information Criterion and the Akaike Information Criterion. The alpha level was set at 0.05 and precision of estimates was indicated by 95% confidence intervals (95% CIs) in all models.

## 3. Results

### 3.1. Sample characteristics

The 636 participants at baseline (T1) identified mostly as female (84.1%) and reported a mean age of 39.5 years (SD = 16.11, range 18–88; Table 1). Participants predominantly held a degree from secondary school (43.7%) or higher education (43.4%). A total of 15.4% were undergoing education, 13.2% were working in a healthcare setting, and 18.1% were unemployed. The majority were married or in a steady relationship (59.1%), had no children (62.6%), and lived together with at least one additional person in the same household (70%). At baseline, 231 (36.3%) individuals reported a diagnosed mental disorder, and four (0.6%) stated a covid-19 infection confirmed by formal testing. At the last assessment (T5), the infection rate with covid-19 increased to 31.5%.

### 3.2. Lockdown measures and care work during the pandemic

The participants’ responses on the perceived lockdown measures at each assessment (T1-T5) were assessed to account for varying regulations in the federal states of Germany (see Supplementary Table S2 for more details). On average, five to seven restrictive measures out of ten were reported at T1, T3, and T4, i.e., during the two lockdown periods. Two measures on average were stated at T2, while little to no measures were reported at T5.

Supplementary Table S3 displays the average responses of a subset of participants at T1 (*n* = 88, 87.5% female), who lived together with their children and working partners. Of these, 63 (71.6%) parents were affected by school or daycare closures, and 31 (54.4%) felt more responsible for childcare during the last weeks compared to their partners. Participants indicated not working less due to childcare (60.7%), but were concerned that the closure of schools and daycare centers would interfere with their work (61.3%).

### 3.3. Longitudinal changes in mental health outcomes across two years of the pandemic

**Figure 2:**
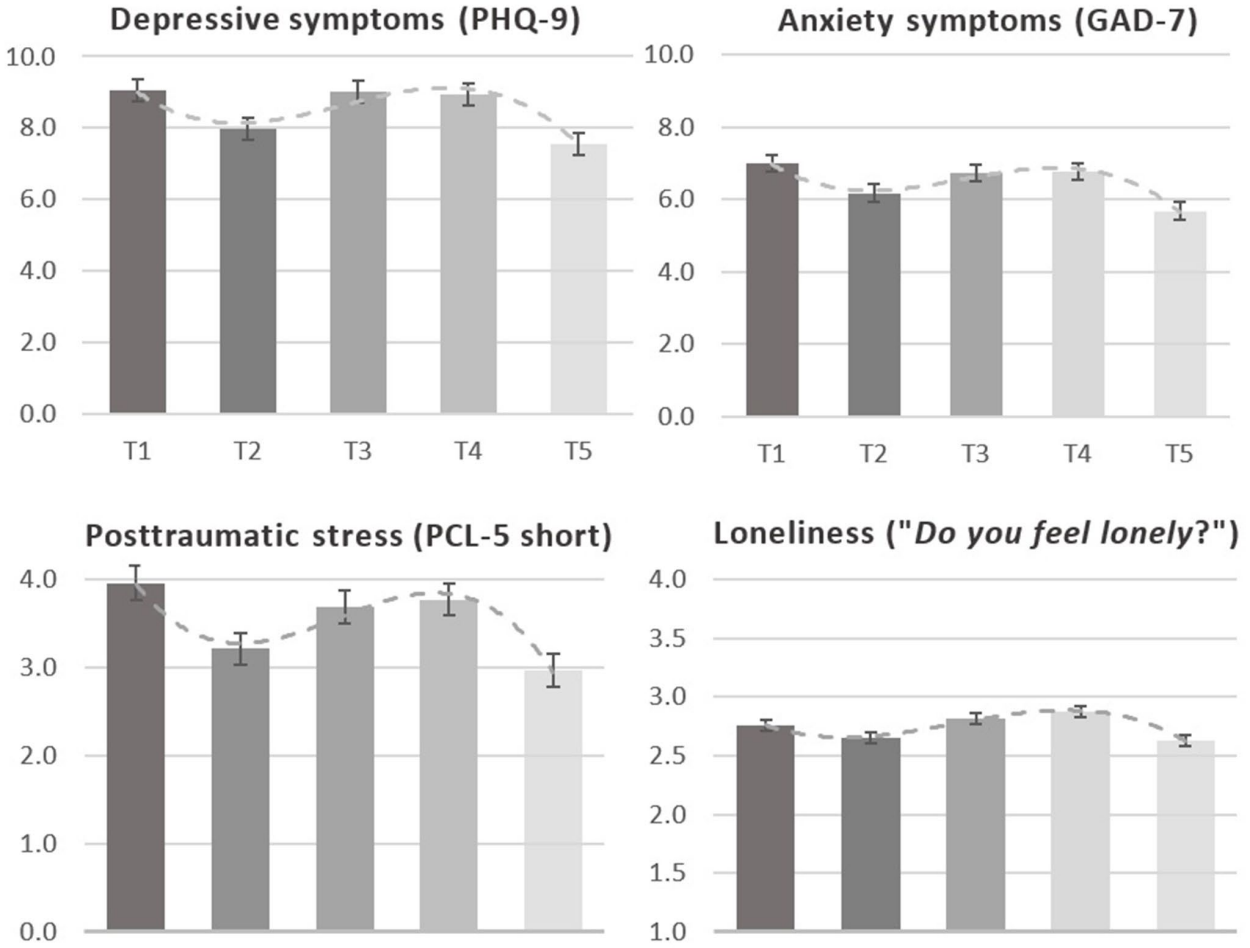
Means, standard errors, and time-cubed trends of mental health outcomes across 2 years of the COVID-pandemic. Scales of the PHQ-9 and GAD-7 range from 0 “not all at” to 3 “nearly every day” (mean scores PHQ-9 0–27; GAD-7 0–21). Scale of posttraumatic stress ranges from 0 “not at all” to 4 “extremely” (mean scores 0–15), rating of loneliness ranges from 1 “never” to 5 “frequently” (mean scores 0–5).

Figure 2 displays the descriptive analyses of the four mental health outcomes across two years of the COVID-pandemic (see Supplementary Tables S4–S7 for additional data). On average, depressive symptoms, anxiety symptoms, posttraumatic stress, and perceived loneliness showed wavelike courses during the pandemic. The highest mean symptom scores, except for perceived loneliness, exhibited during the national lockdowns at T1, and again at T3 and T4. The lowest scores emerged at T2 and T5. Loneliness scores demonstrated delayed average peaks at T3 and T4, i.e., during the second lockdown in Germany.

#### 3.3.1. Longitudinal changes of depression (PHQ-9) and anxiety (GAD-7) scores

Moderate-to-severe symptoms of depression (PHQ-9 > 10) shifted throughout the pandemic with the highest rate at T1 (35.1%), peaking again at T3 (34.3%), and the lowest at T5 (27.8%; 29.2% at T2, 32.9% at T4; Supplementary Table S4). Moderate-to-severe symptoms of anxiety (GAD-7 > 10) showed the highest rate at T1 (25.2%), another peak at T4 (23.5%), and the lowest at T5 (17.7%) (20.3% at T2, 21.7% at T3; Supplementary Table S5).

Table 2 contains the mixed effects modeling for depressive symptoms and anxiety symptoms. The unconditional model included time, time-squared, time-cubed as fixed effects, and time as random effects for each outcome. Coefficients of the fixed effects showed an estimated initial PHQ-9 mean score of 8.96 (8.45, 9.48) and significant linear, quadratic, and cubic trends. Overall, this indicates a decrease in PHQ-9 scores from T1 to T5, while symptoms initially increased from T2 to T4 and subsequently decreased to T5. The estimated GAD-7 mean score at T1 was 6.95 (6.53, 7.37), and significant linear, quadratic, and cubic trends indicate overall decreased anxiety symptoms across the pandemic. However, GAD-7 scores first increased from T2 to T3 followed by a decrease from T3 to T5.

**Table 2:** Mixed unconditional and conditional modeling of symptoms of depression (PHQ-9) and anxiety (GAD-7).

Fixed effects	Unconditional model	*p* value	Conditional model	*p* value
	Estimate (95% CI)^a^		Estimate (95% CI)^a^	
Depressive symptoms				
Intercept	8.96 (8.45–9.48)	<0.001	11.49 (9.76–13.23)	<0.001
Time^b^	−3.52 (−5.60 to −1.44)	<0.001	−3.45 (−5.54 to −1.36)	0.001
Time^2^	8.31 (4.38–12.23)	<0.001	8.29 (4.34–12.24)	<0.001
Time^3^	−3.65 (−5.23 to −2.07)	<0.001	−3.66 (−5.25 to −2.07)	<0.001
Initial age	–		−0.12 (−0.14 to −0.09)	<0.001
Gender	–		2.28 (0.97–3.58)	<0.001
AIC	12,648		12,166	
BIC	12,671		12,189	
Anxiety symptoms				
Intercept	6.95 (6.53–7.37)	<0.001	8.50 (7.06–9.93)	<0.001
Time^b^	−2.86 (−4.68 to −1.04)	0.002	−2.90 (−4.70 to −1.02)	0.002
Time^2^	6.44 (3.00–9.88)	<0.001	6.62 (3.15–10.09)	<0.001
Time^3^	−2.79 (−4.17 to −1.41)	<0.001	−2.89 (−4.29 to −1.50)	<0.001
Initial age	–		−0.07 (−0.10 to −0.05)	<0.001
Gender	–		1.54 (0.47–2.62)	0.005
AIC^c^	11,943		11,524	
BIC^d^	11,966		11,547	

a 95% confidence intervals are in square brackets.

b Coefficients time, time2, and time3 as time-structured predictors with baseline value of zero and consecutive months/12 indicate linear, quadratic, and cubic trends for rates of change.

c AIC, Akaike information Criterion.

d BIC, Bayesian information Criterion.

#### 3.3.2. Associations with depression (PHQ-9) and anxiety (GAD-7) initial scores and longitudinal changes

The conditional mixed effects models also included the time-invariant covariates gender and initial age and showed similar significant linear, quadratic, and cubic trends for PHQ-9 and GAD-7 scores over time (Table 2). At baseline (T1), older participants displayed lower PHQ-9 [*B* = −0.12 (−0.14, −0.09)] and GAD-7 scores [*B* = −0.07 (−0.10, −0.05)], compared to younger adults. In addition, female participants experienced higher initial PHQ-9 [*B* = 2.28 (0.97, 3.58)] and GAD-7 scores [*B* = 1.54 (0.47, 2.62)], compared to males.

**Figure 3:**
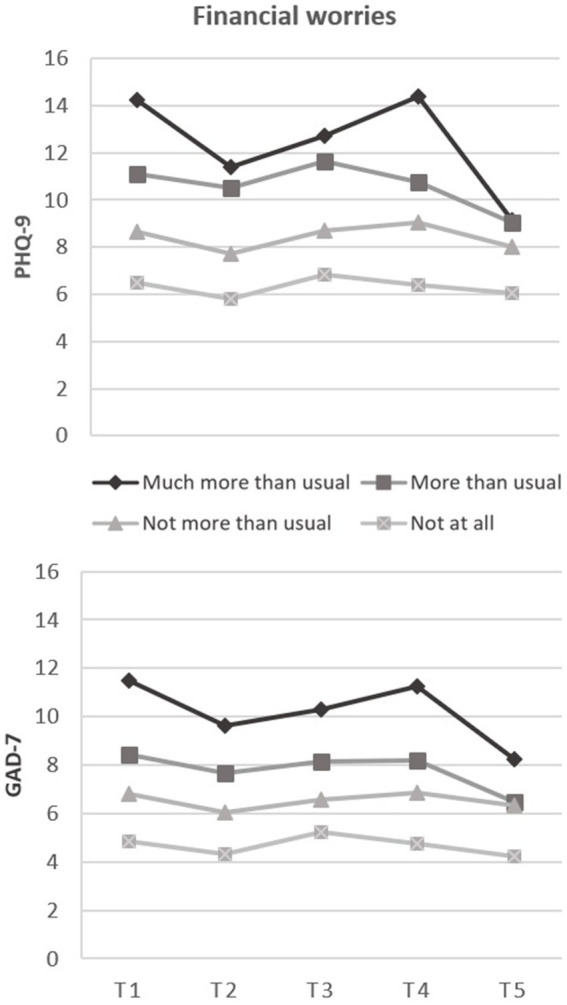
Associations between initial financial worries during the last four weeks and mean trajectories of depressive (PHQ-9) and anxiety symptoms (GAD-7) during two years of the pandemic.

Furthermore, greater PHQ-9 scores at baseline (T1) adjusted by initial age and gender were associated with loneliness [*B* = 2.26 (1.99, 2.52)], contamination fear [*B* = 0.12 (0.07, 0.17)], and financial worries [*B* = 2.39 (1.89, 2.90)]. However, these variables were not associated with changes in PHQ-9 scores across the pandemic, except for financial worries. Greater financial worries at T1 were associated with overall greater decreased PHQ-9 scores in the course of the pandemic, as indicated by significant linear [*B* = −3.88 (−6.21, −1.56)], quadratic [*B* = 7.20 (2.78, 11.61)], and cubic interaction terms [*B* = −2.97 (−4.75, −1.19); see Figure 3].

Similarly, greater initial GAD-7 scores adjusted by initial age and gender were associated with loneliness [*B* = 1.44 (1.20, 1.67)], contamination fear [*B* = 0.10 (0.05, 0.14)], and financial worries [*B* = 2.01 (1.58, 2.43)]. Decreasing GAD-7 scores during the pandemic were associated with greater financial worries at T1, as indicated by significant linear [*B* = −2.91 (−4.94, −0.87)], quadratic [*B* = 5.03 (1.15, 8.90)], and cubic interaction terms [*B* = −2.01 (−3.57, −0.45); Figure 3]. No associations were found between the predictors having children and student status, respectively, and PHQ-9 or GAD-7 scores.

#### 3.3.3. Longitudinal changes of posttraumatic stress (PCL-5) and loneliness scores

Table 3 entails the unconditional and conditional mixed effects models for the PCL-5 and loneliness scores. Coefficients of the fixed effects showed significant linear, quadratic, and cubic trends for the PCL-5 scores, indicating an overall decrease in posttraumatic stress symptoms across the pandemic. For loneliness, coefficients included significant quadratic and cubic trends, suggesting averaged decreased loneliness during the pandemic. Entering the time-invariant covariates gender and initial age, the conditional models showed similar trends for the PCL-5 and loneliness scores compared to the unconditional models. Females compared to male participants, and younger adults compared to older participants, experienced higher levels of posttraumatic stress and loneliness, respectively.

**Table 3:** Mixed unconditional and conditional modeling of posttraumatic stress (short-form PCL-5) and loneliness (“Do you feel lonely?”).

Fixed effects	Unconditional model	*p* value	Conditional model	*p* value
	Estimate (95% CI)^a^		Estimate (95% CI)^a^	
Posttraumatic stress				
Intercept	3.92 (3.66–4.19)	<0.001	4.77 (3.85–5.70)	<0.001
Time^b^	−2.95 (−4.18 to −1.73)	<0.001	−3.03 (−4.28 to −1.79)	<0.001
Time^2^	6.24 (3.93–8.56)	<0.001	6.39 (4.04–8.74)	<0.001
Time^3^	−2.66 (−3.59 to −1.73)	<0.001	−2.72 (−3.66 to −1.77)	<0.001
Initial age	–		−0.04 (−0.05 to −0.02)	<0.001
Gender	–		0.67 (−0.02 to 1.36)	0.058
AIC	10,235		9,859	
BIC	10,258		9,917	
Loneliness				
Intercept	2.75 (2.66–2.84)	<0.001	3.18 (2.87–3.49)	<0.001
Time^b^	−0.37 (−0.82 to 0.08)	0.110	−0.32 (−0.78 to 0.14)	0.169
Time^2^	1.28 (0.42–2.14)	0.003	1.18 (0.31–2.05)	0.008
Time^3^	−0.60 (−0.94 to −0.25)	<0.001	−0.56 (−0.91 to −0.21)	0.002
Initial age	–		−0.02 (−0.02 to −0.01)	<0.001
Gender	–		0.24 (0.01–0.47)	0.044
AIC^c^	5,715		5,518	
BIC^d^	5,738		5,541	

a 95% confidence intervals are in square brackets.

b Coefficients time, time2, and time3 as time-structured predictors with baseline value of zero and consecutive months/12 indicate linear, quadratic, and cubic trends for rates of change.

c AIC, Akaike information Criterion.

d BIC, Bayesian information Criterion.

## 4. Discussion

### 4.1. Summary and interpretation of findings

This five-wave longitudinal study examined the temporal dynamics of mental health outcomes among *N* = 636 individuals across two pandemic years in Germany. On average, symptoms of depression, anxiety, posttraumatic stress, and loneliness declined from May 2020 (T1) to April 2022 (T5). All mental health outcomes peaked during the two national lockdown phases in Germany (at T1, T3, and T4) and dropped during the easing phases (at T2 and T5). Symptom scores were lowest at T5, respectively, where infection rates were high, but all remaining restrictions were lifted. At T1, higher scores of depression and anxiety were evident in female and younger participants. In addition, higher initial depressive and anxiety scores were associated with increased levels of contamination fear, loneliness, and financial worries. Participants reporting greater initial financial worries showed higher depressive and anxiety symptom levels at each assessment time point but also showed greater symptom decreases in anxiety and depression across the pandemic.

The fluctuating depressive and anxiety symptoms during the pandemic are in line with several previous findings of longitudinal studies: during the first months of the pandemic, higher levels of anxiety and depression have been repetitively reported across the globe [e.g., (11, 13, 46)], which mostly diminished as the pandemic progressed and restrictive measures were eased (10, 13). Covering the later phases of the pandemic (13, 17, 22, 23, 47), for instance, the COMET longitudinal study on the French general population found varying scores of depression, anxiety, and posttraumatic stress symptoms spanning 1 year of the pandemic, consistent with our findings (17). Symptoms were higher during the lockdown phases and lower during the easing phases, which has also been reported for anxiety and depressive symptoms in a German sample during the same period (23). In our study, scores of depression, anxiety, and posttraumatic stress were highest at the first assessment, i.e., at the end of the first lockdown, and increased almost as high during the second lockdown period. This is somewhat comparable to findings from Hettich et al. (22), showing declined symptoms of anxiety and depression during the second lockdown compared to the end of the first lockdown in Germany. Conversely, they found decreased levels of life satisfaction during both periods and large increases in loneliness from the first to the second lockdown. Regarding loneliness, our data showed decreases after the first lockdown as reported earlier (17, 48), and similar delayed peaks during the second lockdown (22, 49, 50). Lonely individuals were likely to perceive changes in the quantity or quality in social connections (51) more drastically during the lockdown periods since reduced social contacts were a key element of the containment measures in place. The results also suggest that containment measures might have a cumulative impact on the levels of perceived loneliness (18), as indicated by the average increase in loneliness during the subsequent and long-lasting lockdown in Germany.

Overall, our findings on depression, anxiety, posttraumatic stress, and loneliness scores support our hypotheses and the notion that prolonged and repeated lockdown measures were associated with increased mental burden (12, 52). This also translates into the vulnerability-stress model (53), suggesting increased vulnerability for psychopathology during demanding phases in life with multifaced stressors, such as a pandemic. In addition, our results on the long-term effects of the pandemic are consistent with the assumption that most individuals were resilient and recovered from the lockdown periods (47, 54). This becomes evident by the overall declining courses during the easing phases. To date, longitudinal data on mental health during the second pandemic year is still scarce, limiting the comparability of the complete survey period of our study with other studies. Contrary to previous concerns regarding delayed increased symptomatology (55), we found the lowest scores of depression, anxiety, and posttraumatic stress at the latest assessment in April 2022 (T5). This result is mirrored by a recently published study reporting declined anxiety and depressive symptoms in an Italian sample after two pandemic years (56). The extent of the lockdown measures implemented in Italy was alternately stricter and looser, depending on the infectious situation, as comparable to regulations in Germany and many other European countries (57).

Notably, we detected that prevalence rates of moderate-to-severe depression and anxiety declined by 7.3 and 7.5 points from baseline to T5, although infections with the prevailing SARS-CoV-2 Omicron variant reached a dramatic high at this time. At least at this time point, our findings contradict previous notions that the infection rate can be considered a pivotal factor in predicting mental burden during the pandemic (23). The highly transmissible Omicron variant was generally perceived as less life-threatening, also by medical professionals (58), possibly associated with less psychological distress. The declining symptoms at T5 might be also explained via the easing of all remaining containment measures at T5 in Germany. Simultaneously, the full vaccination coverage was high in our sample (93.1%) and in Germany in general [75.6%; (59)], perhaps further buffering threat-related symptoms (60).

However, we also identified individuals with dispositions associated with greater mental health risks during the pandemic, thereby paralleling the current body of evidence on both female and younger individuals (13, 17, 47, 61, 62). Women are more likely to be healthcare workers, work in child care, and report internalizing disorders in general than men (63), potentially increasing the mental burden during the pandemic. In general, assessment and reporting biases in self-reporting due to societal gender roles (64) may play a role but also underlying biological vulnerabilities, including genetic, neurologic, and hormonal factors (65–68).

Adjusted by gender and age, contamination fear and loneliness were uniquely associated with higher anxiety and depressive symptoms, which replicates previous research on OCD symptomatology (19, 69) and loneliness (17, 22, 70) during the pandemic. However, we did not find associations with changes in mental health, except for financial worries. Individuals worried about their financial situation reported higher levels of depression and anxiety at each time point, as reported previously in other countries (24, 71), whereas symptom levels decreased steeper over time. Individuals with higher financial worries likely responded more adverse to the pandemic and lockdown at baseline, allowing more room for improvement in the course of the pandemic. Those with greater financial worries were likely to initially respond more negatively to the pandemic, leaving more room for improvement over time. Possibly the continuing pandemic was less dire than expected. This is supported by the insecure positions or job losses during the initial lockdown in Germany (72), which were successively compensated by enormous government subsidies (73). This may have contributed to greater financial security and alleviated the symptoms of affected individuals in the long term.

In contrast to our hypotheses, student status was not related to the initial anxiety and depressive symptoms or changes during the pandemic after controlling for associations with younger age and female gender. This also contrasts prior findings, which demonstrated increased mental strain in college and university students compared to the general population (2, 13, 61, 74). In addition, prior studies focusing on individuals with children suggested an increased risk for deteriorated mental health during the pandemic compared to pre-pandemic years, which was not evident in the present study. In our data, however, most participants living with their children and working partners reported being stressed by school or daycare closures. This is consistent with previous findings that shed light on the challenging position of families during the pandemic in Germany (75).

### 4.2. Strengths and limitations

This longitudinal cohort study has several strengths: we examined the mental health courses in a German adult sample across five waves during two years of the COVID-19 pandemic, which exceeds the temporal scope of most previous studies. We emphasized several facets of mental health, i.e., symptoms of depression, anxiety, posttraumatic stress, and loneliness, and we assessed potentially associated factors with symptom severity and change. However, limitations should also be considered: The sample was recruited using convenience sampling and is mainly comprised of female and highly educated participants. The results are thus not representative of the general German population, which should be considered when interpreting the results (65). In addition, the attrition rate was 33% after one year and 66% after two years of data collection. Although reliable and valid, self-rated questionnaires were used in this online study, potentially distorting the results. To fully capture the pandemic’s impact on mental health, a formal diagnosis of mental disorders, help-seeking behavior, and positive indicators such as quality of life should additionally be monitored in the long term. Lastly, the data collection began in the early phase of the pandemic and precluded inferences on mental health changes relative to pre-pandemic years. Yet, our study design included different lockdown and easing periods instead, enabling comparisons between mental health states across two pandemic years.

## 5. Conclusion

Examining the long-term course of mental health, this study indicated overall decreasing but dynamic levels of mental burden in parallel to the pandemic situation in Germany. Specifically, individuals showed increased symptoms of depression, anxiety, posttraumatic stress, and loneliness in times with pronounced lockdown phases, and decreased symptoms during the easing phases. This highlights the need for evidence-based support services – especially at the onset and during lockdown periods – to better address mental health challenges related to fear of infectious events, social distancing, or isolation. One option involves accessible online-based cognitive behavioral training and treatment, demonstrating improved anxiety and depressive symptoms across twelve randomized controlled trials conducted during the pandemic (76). Although most individuals adapted and recovered during the two-year pandemic, particularly vulnerable subgroups including younger, female, and financially insecure individuals should be monitored long-term and targeted for evidence-based services and interventions as needed.

## Data availability statement

The raw data supporting the conclusions of this article will be made available by the authors, without undue reservation.

## Ethics statement

The studies involving human participants were reviewed and approved by the Scientific and Ethical Review Board of Vrije Universiteit Amsterdam (VCWE-2020-077) and the Department of Education and Psychology at Freie Universität Berlin (023/2020). The patients/participants provided their written informed consent to participate in this study.

## Author contributions

MW, SB, MS, MP, IP, BR, CK, and SS contributed to the design of the study and to the acquisition, analysis, or interpretation of the data. MW performed the statistical analysis and wrote the first draft of the manuscript. All authors contributed to the article and approved the submitted version.

## Conflict of interest

The authors declare that the research was conducted in the absence of any commercial or financial relationships that could be construed as a potential conflict of interest.

## Publisher’s note

All claims expressed in this article are solely those of the authors and do not necessarily represent those of their affiliated organizations, or those of the publisher, the editors and the reviewers. Any product that may be evaluated in this article, or claim that may be made by its manufacturer, is not guaranteed or endorsed by the publisher.

## References

[1] Wirkner J Christiansen H Knaevelsrud C Lüken U Wurm S Schneider S. Mental health in times of the COVID-19 pandemic. Eur Psychol. (2021) 26:310–22. doi: 10.1027/1016-9040/a000465

[2] Dragioti E Li H Tsitsas G Lee KH Choi J Kim J. A large-scale meta-analytic atlas of mental health problems prevalence during the COVID-19 early pandemic. J Med Virol. (2022) 94:1935–49. doi: 10.1002/jmv.27549, PMID: 34958144 PMC9015528

[3] Leung CM Ho MK Bharwani AA Cogo-Moreira H Wang Y Chow MS. Mental disorders following COVID-19 and other epidemics: a systematic review and meta-analysis. Transl Psychiatry. (2022) 12:205. doi: 10.1038/s41398-022-01946-6, PMID: 35581186 PMC9110635

[4] Luo M Guo L Yu M Jiang W Wang H. The psychological and mental impact of coronavirus disease 2019 (COVID-19) on medical staff and general public – a systematic review and meta-analysis. Psychiatry Res. (2020) 291:113190. doi: 10.1016/j.psychres.2020.113190, PMID: 32563745 PMC7276119

[5] Nochaiwong S Ruengorn C Thavorn K Hutton B Awiphan R Phosuya C. Global prevalence of mental health issues among the general population during the coronavirus disease-2019 pandemic: a systematic review and meta-analysis. Sci Rep. (2021) 11:10173. doi: 10.1038/s41598-021-89700-8, PMID: 33986414 PMC8119461

[6] Salari N Hosseinian-Far A Jalali R Vaisi-Raygani A Rasoulpoor S Mohammadi M. Prevalence of stress, anxiety, depression among the general population during the COVID-19 pandemic: a systematic review and meta-analysis. Glob Health. (2020) 16:57. doi: 10.1186/s12992-020-00589-w, PMID: 32631403 PMC7338126

[7] Vindegaard N Benros ME. COVID-19 pandemic and mental health consequences: systematic review of the current evidence. Brain Behav Immun. (2020) 89:531–42. doi: 10.1016/j.bbi.2020.05.048, PMID: 32485289 PMC7260522

[8] Wu T Jia X Shi H Niu J Yin X Xie J. Prevalence of mental health problems during the COVID-19 pandemic: a systematic review and meta-analysis. J Affect Disord. (2021) 281:91–8. doi: 10.1016/j.jad.2020.11.117, PMID: 33310451 PMC7710473

[9] Kunzler AM Röthke N Günthner L Stoffers-Winterling J Tüscher O Coenen M. Mental burden and its risk and protective factors during the early phase of the SARS-CoV-2 pandemic: systematic review and meta-analyses. Glob Health. (2021) 17:34. doi: 10.1186/s12992-021-00670-y, PMID: 33781283 PMC8006628

[10] Richter D Riedel-Heller S Zürcher SJ. Mental health problems in the general population during and after the first lockdown phase due to the SARS-Cov-2 pandemic: rapid review of multi-wave studies. Epidemiol Psychiatr Sci. (2021) 30:e27. doi: 10.1017/S2045796021000160, PMID: 33685551 PMC7985862

[11] Schafer KM Lieberman A Sever AC Joiner T. Prevalence rates of anxiety, depressive, and eating pathology symptoms between the pre-and peri-COVID-19 eras: a meta-analysis. J Affect Disord. (2022) 298:364–72. doi: 10.1016/j.jad.2021.10.115, PMID: 34740748 PMC8593520

[12] Prati G Mancini AD. The psychological impact of COVID-19 pandemic lockdowns: a review and meta-analysis of longitudinal studies and natural experiments. Psychol Med. (2021) 51:201–11. doi: 10.1017/S0033291721000015, PMID: 33436130 PMC7844215

[13] Robinson E Sutin AR Daly M Jones A. A systematic review and meta-analysis of longitudinal cohort studies comparing mental health before versus during the COVID-19 pandemic in 2020. J Affect Disord. (2022) 296:567–76. doi: 10.1016/j.jad.2021.09.098, PMID: 34600966 PMC8578001

[14] Sun Y Wu Y Fan S Dal Santo T Li L Jiang X. Comparison of mental health symptoms before and during the covid-19 pandemic: evidence from a systematic review and meta-analysis of 134 cohorts. BMJ. (2023) 380:e074224. doi: 10.1136/bmj-2022-074224, PMID: 36889797 PMC9992728

[15] Witteveen AB Young SY Cuijpers P Ayuso-Mateos JL Barbui C Bertolini F. COVID-19 and common mental health symptoms in the early phase of the pandemic: an umbrella review of the evidence. PLoS Med. (2023) 20:e1004206. doi: 10.1371/journal.pmed.1004206, PMID: 37098048 PMC10129001

[16] Gémes K Bergström J Papola D Barbui C Lam AI Hall BJ. Symptoms of anxiety and depression during the COVID-19 pandemic in six European countries and Australia – differences by prior mental disorders and migration status. J Affect Disord. (2022) 311:214–23. doi: 10.1016/j.jad.2022.05.082, PMID: 35598751 PMC9119165

[17] Moulin F Jean F Melchior M Patanè M Pinucci I Sijbrandij M. Longitudinal impact of the COVID19 pandemic on mental health in a general population sample in France: evidence from the COMET study. J Affect Disord. (2023) 320:275–83. doi: 10.1016/j.jad.2022.09.142 36191642 PMC9525187

[18] Laham S Bertuzzi L Deguen S Hecker I Melchior M Patanè M. Impact of longitudinal social support and loneliness trajectories on mental health during the COVID-19 pandemic in France. Int J Environ Res Public Health. (2021) 18:12677. doi: 10.3390/ijerph182312677, PMID: 34886402 PMC8656819

[19] Tarsitani L Pinucci I Tedeschi F Patanè M Papola D Palantza C. Resilience of people with chronic medical conditions during the COVID-19 pandemic: a 1-year longitudinal prospective survey. BMC Psychiatry. (2022) 22:633. doi: 10.1186/s12888-022-04265-8, PMID: 36183067 PMC9525930

[20] Daly M Robinson E. Psychological distress associated with the second COVID-19 wave: prospective evidence from the UK household longitudinal study. J Affect Disord. (2022) 310:274–8. doi: 10.1016/j.jad.2022.05.025, PMID: 35568319 PMC9091072

[21] Del-Valle MV López-Morales H Gelpi-Trudo R Poó FM García MJ Yerro-Avincetto M. More than a year of pandemic: longitudinal assessment of anxiety and depressive symptoms in the argentine general population during the COVID-19 outbreak. Stress Health. (2022) 38:1070–9. doi: 10.1002/smi.3163, PMID: 35574626 PMC9348304

[22] Hettich N Entringer TM Kroeger H Schmidt P Tibubos AN Braehler E. Impact of the COVID-19 pandemic on depression, anxiety, loneliness, and satisfaction in the German general population: a longitudinal analysis. Soc Psychiat Epidemiol. (2022) 57:2481–90. doi: 10.1007/s00127-022-02311-0, PMID: 35680681 PMC9181932

[23] Bendau A Asselmann E Plag J Petzold MB Ströhle A. 1.5 years pandemic – psychological burden over the course of the COVID-19 pandemic in Germany: a nine-wave longitudinal community study. J Affect Disord. (2022) 319:381–7. doi: 10.1016/j.jad.2022.09.105, PMID: 36162668 PMC9507788

[24] Matsubayashi T Ishikawa Y Ueda M. Economic crisis and mental health during the COVID-19 pandemic in Japan. J Affect Disord. (2022) 306:28–31. doi: 10.1016/j.jad.2022.03.037, PMID: 35306120 PMC8924027

[25] Dong E Du H Gardner L. An interactive web-based dashboard to track COVID-19 in real time. Lancet Infect Dis. (2020) 20:533–4. doi: 10.1016/S1473-3099(20)30120-1, PMID: 32087114 PMC7159018

[26] Bundesregierung. (2020). Besprechung der Bundeskanzlerin mit den Regierungschefinnen und Regierungschefs der Länder vom 22.03.2020 (Meeting of the Chancellor with the Heads of Government of the Federal States and Länder on 22.03.2020) Available at: https://www.bundeskanzler.de/bk-de/leichte-sprache/6-mai-2020-regeln-zum-corona-virus-1755252 (Accessed February 05, 2023).

[27] Bundesregierung. (2020). 6. Mai 2020: Regeln zum Corona-virus (6 may 2020, rules on the Corona virus). Available at: https://www.bundeskanzler.de/bk-de/leichte-sprache/6-mai-2020-regeln-zum-corona-virus-1755252 (Accessed February 05, 2023).

[28] Bundesregierung. (2020). Bund-Länder-Beschluss zur Corona-Pandemie. “Wir müssen handeln – und zwar jetzt” (Federal-Länder decision on the Corona pandemic. “we must act – and act now”). Available at: https://www.bundesregierung.de/breg-de/suche/bund-laender-beschluss-1804936 (Accessed February 05, 2023).

[29] Bundesregierung. (2020). Die Regelungen im Überblick. “Wir sind zum Handeln gezwungen” (The regulations at a glance. “We are forced to act”). Available at: https://www.bundesregierung.de/breg-de/themen/coronavirus/merkel-beschluss-weihnachten-1827396 (Accessed February 05, 2023).

[30] Bundesregierung. (2022). Bund-Länder-Konferenz zur Corona-situation. Wir treten in eine neue phase der Pandemie ein (Federal-Länder Conference on the Corona situation. We enter a new phase of the pandemic). Available at: https://www.bundeskanzler.de/bk-de/suche/bund-laender-treffen-pandemie-2017528 (Accessed February 05, 2023).

[31] Elm v E Altman DG Egger M Pocock SJ Gøtzsche PC Vandenbroucke JP. The strengthening the reporting of observational studies in epidemiology (STROBE) statement: guidelines for reporting observational studies. Lancet. (2007) 370:1453–7. doi: 10.1016/S0140-6736(07)61602-X 18064739

[32] Kroenke K Spitzer RL Williams JB. The PHQ-9: validity of a brief depression severity measure. J Gen Intern Med. (2001) 16:606–13. doi: 10.1046/j.1525-1497.2001.016009606.x, PMID: 11556941 PMC1495268

[33] Kocalevent R-D Hinz A Brähler E. Standardization of the depression screener patient health questionnaire (PHQ-9) in the general population. Gen Hosp Psychiatry. (2013) 35:551–5. doi: 10.1016/j.genhosppsych.2013.04.006, PMID: 23664569

[34] Löwe B Decker O Müller S Brähler E Schellberg D Herzog W. Validation and standardization of the generalized anxiety disorder screener (GAD-7) in the general population. Med Care. (2008) 46:266–74. doi: 10.1097/MLR.0b013e318160d093, PMID: 18388841

[35] Spitzer RL Kroenke K Williams JB Löwe B. A brief measure for assessing generalized anxiety disorder: the GAD-7. Arch Intern Med. (2006) 166:1092–7. doi: 10.1001/archinte.166.10.1092 16717171

[36] Zuromski KL Ustun B Hwang I Keane TM Marx BP Stein MB. Developing an optimal short-form of the PTSD checklist for DSM-5 (PCL-5). Depress Anxiety. (2019) 36:790–800. doi: 10.1002/da.22942, PMID: 31356709 PMC6736721

[37] Price M Szafranski DD van Stolk-Cooke K Gros DF. Investigation of abbreviated 4 and 8 item versions of the PTSD checklist 5. Psychiatry Res. (2016) 239:124–30. doi: 10.1016/j.psychres.2016.03.014, PMID: 27137973

[38] Mund M Maes M Drewke PM Gutzeit A Jaki I Qualter P. Would the real loneliness please stand up? The validity of loneliness scores and the reliability of single-item scores. Assessment. (2022) 30:1226–48. doi: 10.1177/10731911221077227, PMID: 35246009 PMC10149889

[39] Corp IBM. IBM SPSS statistics for windows (version 29.0). Armonk, NY: IBM Corp (2022). Available at: https://www.ibm.com/products/spss-statistics

[40] Laird NM Ware JH. Random-effects models for longitudinal data. Biometrics. (1982) 38:963. doi: 10.2307/2529876 7168798

[41] Singer JD Willett JB. Applied longitudinal data analysis. New York, NY: Oxford University Press (2003).

[42] Twisk J Boer M d Vente W d Heymans M. Multiple imputation of missing values was not necessary before performing a longitudinal mixed-model analysis. J Clin Epidemiol. (2013) 66:1022–8. doi: 10.1016/j.jclinepi.2013.03.017 23790725

[43] Curran PJ Obeidat K Losardo D. Twelve frequently asked questions about growth curve modeling. J Cogn Dev. (2010) 11:121–36. doi: 10.1080/15248371003699969 21743795 PMC3131138

[44] Burns GL Keortge SG Formea GM Sternberger LG. Revision of the Padua inventory of obsessive compulsive disorder symptoms: distinctions between worry, obsessions, and compulsions. Behav Res Ther. (1996) 34:163–73. doi: 10.1016/0005-7967(95)00035-6, PMID: 8741724

[45] Gönner S Ecker W Leonhart R. The Padua inventory: do revisions need revision? Assessment. (2010) 17:89–106. doi: 10.1177/1073191109342189, PMID: 19745211

[46] Pierce M Hope H Ford T Hatch S Hotopf M John A. Mental health before and during the COVID-19 pandemic: a longitudinal probability sample survey of the UK population. Lancet Psychiatry. (2020) 7:883–92. doi: 10.1016/S2215-0366(20)30308-4, PMID: 32707037 PMC7373389

[47] Fancourt D Steptoe A Bu F. Trajectories of anxiety and depressive symptoms during enforced isolation due to COVID-19 in England: a longitudinal observational study. Lancet Psychiatry. (2021) 8:141–9. doi: 10.1016/s2215-0366(20)30482-x, PMID: 33308420 PMC7820109

[48] Entringer TM Gosling SD. Loneliness during a Nationwide lockdown and the moderating effect of extroversion. Soc Psychol Personal Sci. (2022) 13:769–80. doi: 10.1177/19485506211037871

[49] Weber M Schulze L Bolzenkötter T Niemeyer H Renneberg B. Mental health and loneliness in university students during the COVID-19 pandemic in Germany: a longitudinal study. Front Psych. (2022) 13:848645. doi: 10.3389/fpsyt.2022.848645, PMID: 35492687 PMC9051079

[50] Benke C Asselmann E Entringer TM Pané-Farré CA. The role of pre-pandemic depression for changes in depression, anxiety, and loneliness during the COVID-19 pandemic: results from a longitudinal probability sample of adults from Germany. Eur Psychiatry. (2022) 65:e76. doi: 10.1192/j.eurpsy.2022.2339, PMID: 36325825 PMC9706309

[51] Hawkley LC Cacioppo JT. Loneliness matters: a theoretical and empirical review of consequences and mechanisms. Ann Behav Med. (2010) 40:218–27. doi: 10.1007/s12160-010-9210-8, PMID: 20652462 PMC3874845

[52] Henssler J Stock F Bohemen J Walter H Heinz A Brandt L. Mental health effects of infection containment strategies: quarantine and isolation—a systematic review and meta-analysis. Eur Arch Psychiatry Clin Neurosci. (2021) 271:223–34. doi: 10.1007/s00406-020-01196-x, PMID: 33025099 PMC7538183

[53] Ingram J Maciejewski G Hand CJ. Changes in diet, sleep, and physical activity are associated with differences in negative mood during COVID-19 lockdown. Front Psychol. (2020) 11:588604. doi: 10.3389/fpsyg.2020.588604, PMID: 32982903 PMC7492645

[54] Penninx BW Benros ME Klein RS Vinkers CH. How COVID-19 shaped mental health: from infection to pandemic effects. Nat Med. (2022) 28:2027–37. doi: 10.1038/s41591-022-02028-2, PMID: 36192553 PMC9711928

[55] COVID-19 Mental Disorders Collaborators. Global prevalence and burden of depressive and anxiety disorders in 204 countries and territories in 2020 due to the COVID-19 pandemic. Lancet. (2021) 398:1700–12. doi: 10.1016/S0140-6736(21)02143-7, PMID: 34634250 PMC8500697

[56] Mazza C Ricci E Colasanti M Cardinale A Bosco F Biondi S. How has COVID-19 affected mental health and lifestyle behaviors after 2 years? The third step of a longitudinal study of Italian citizens. Int J Environ Res Public Health. (2022) 20:20. doi: 10.3390/ijerph20010759, PMID: 36613081 PMC9819689

[57] Hale T Angrist N Goldszmidt R Kira B Petherick A Phillips T. A global panel database of pandemic policies (Oxford COVID-19 government response tracker). Nat Hum Behav. (2021) 5:529–38. doi: 10.1038/s41562-021-01079-8, PMID: 33686204

[58] Lorenzo-Redondo R Ozer EA Hultquist JF. Covid-19: is omicron less lethal than delta? BMJ. (2022) 378:o1806. doi: 10.1136/bmj.o1806 35918084

[59] Robert Koch Institut. (2022). Wöchentlicher Lagebericht des RKI zur coronavirus-Krankheit-2019 (COVID-19) [weekly RKI report on the coronavirus disease-2019 (COVID-19)]. Available at: https://www.rki.de/DE/Content/InfAZ/N/Neuartiges_Coronavirus/Situationsberichte/Wochenbericht/Wochenbericht_2022-03-10.pdf?__blob=publicationFile (Accessed January 30, 2023).

[60] Chen S Aruldass AR Cardinal RN. Mental health outcomes after SARS-CoV-2 vaccination in the United States: a national cross-sectional study. J Affect Disord. (2022) 298:396–9. doi: 10.1016/j.jad.2021.10.134, PMID: 34774648 PMC8580571

[61] Xiong J Lipsitz O Nasri F Lui LM Gill H Phan L. Impact of COVID-19 pandemic on mental health in the general population: a systematic review. J Affect Disord. (2020) 277:55–64. doi: 10.1016/j.jad.2020.08.001 32799105 PMC7413844

[62] Tibubos AN Otten D Ernst M Beutel ME. A systematic review on sex-and gender-sensitive research in public mental health during the first wave of the COVID-19 crisis. Front Psych. (2021) 12:712492. doi: 10.3389/fpsyt.2021.712492, PMID: 34603104 PMC8484908

[63] van Loo HM Beijers L Wieling M Jong TR de Schoevers RA Kendler KS. Prevalence of internalizing disorders, symptoms, and traits across age using advanced nonlinear models. Psychol Med (2023) 53:78–87. doi: 10.1017/S0033291721001148, PMID: 33849670 PMC9874996

[64] Giel KE Derntl B. The weaker sex? What we can learn from sex differences in population mental health during and beyond the COVID-19 pandemic. Eur Arch Psychiatry Clin Neurosci. (2022) 272:165–6. doi: 10.1007/s00406-021-01312-5, PMID: 34324033 PMC8319593

[65] Altemus M Sarvaiya N Neill EC. Sex differences in anxiety and depression clinical perspectives. Front Neuroendocrinol. (2014) 35:320–30. doi: 10.1016/j.yfrne.2014.05.004, PMID: 24887405 PMC4890708

[66] Farhane-Medina NZ Luque B Tabernero C Castillo-Mayén R. Factors associated with gender and sex differences in anxiety prevalence and comorbidity: a systematic review. Sci Prog. (2022) 105:003685042211354. doi: 10.1177/00368504221135469 PMC10450496 36373774

[67] Li SH Graham BM. Why are women so vulnerable to anxiety, trauma-related and stress-related disorders? The potential role of sex hormones. Lancet Psychiatry. (2017) 4:73–82. doi: 10.1016/S2215-0366(16)30358-3, PMID: 27856395

[68] Pavlidi P Kokras N Dalla C. Sex differences in depression and anxiety. In: Gibson C. Galea L.a.M. (eds) sex differences in brain function and dysfunction. Curr Top Behav Neurosci. (2023) 62:103–32. doi: 10.1007/7854_2022_375, PMID: 35915385

[69] Grant JE Drummond L Nicholson TR Fagan H Baldwin DS Fineberg NA. Obsessive-compulsive symptoms and the Covid-19 pandemic: a rapid scoping review. Neurosci Biobehav Rev. (2022) 132:1086–98. doi: 10.1016/j.neubiorev.2021.10.039, PMID: 34740755 PMC8570941

[70] Pai N Vella S-L. COVID-19 and loneliness: a rapid systematic review. Aust N Z J Psychiatry. (2021) 55:1144–56. doi: 10.1177/00048674211031489 34256632

[71] Hertz-Palmor N Moore TM Gothelf D DiDomenico GE Dekel I Greenberg DM. Association among income loss, financial strain and depressive symptoms during COVID-19: evidence from two longitudinal studies. J Affect Disord. (2021) 291:1–8. doi: 10.1016/j.jad.2021.04.054, PMID: 34022550 PMC8460400

[72] Liu S Heinzel S Haucke MN Heinz A. Increased psychological distress, loneliness, and unemployment in the spread of COVID-19 over 6 months in Germany. Medicina (Kaunas). (2021) 57:57. doi: 10.3390/medicina57010053, PMID: 33435368 PMC7827929

[73] Federal Ministry of Finance. (2022). Comprehensive pandemic-related assistance for companies and self-employed individuals. Available at: https://www.bundesfinanzministerium.de/Content/EN/Standardartikel/Topics/Priority-Issues/Corona/comprehensive-pandemic-crisis-support.html (Accessed February 22, 2023).

[74] Pappa S Chen J Barnett J Chang A Dong RK Xu W. A systematic review and meta-analysis of the mental health symptoms during the Covid-19 pandemic in Southeast Asia. Psychiatry Clin Neurosci. (2022) 76:41–50. doi: 10.1111/pcn.13306, PMID: 34704305 PMC8661667

[75] Calvano C Engelke L Di Bella J Kindermann J Renneberg B Winter SM. Families in the COVID-19 pandemic: parental stress, parent mental health and the occurrence of adverse childhood experiences—results of a representative survey in Germany. Eur Child Adolesc Psychiatry. (2022) 31:1–13. doi: 10.1007/s00787-021-01739-0 PMC7917379 33646416

[76] Komariah M Amirah S Faisal EG Prayogo SA Maulana S Platini H. Efficacy of internet-based cognitive behavioral therapy for depression and anxiety among global population during the COVID-19 pandemic: a systematic review and Meta-analysis of a randomized controlled trial study. Healthcare (Basel). (2022) 10:1224. doi: 10.3390/healthcare10071224, PMID: 35885751 PMC9315502

